# Young, Bullying, and Connected. Common Pathways to Cyberbullying and Problematic Internet Use in Adolescence

**DOI:** 10.3389/fpsyg.2019.01467

**Published:** 2019-07-04

**Authors:** Antonella Brighi, Damiano Menin, Grace Skrzypiec, Annalisa Guarini

**Affiliations:** ^1^Faculty of Education, Campus of Bressanone, Free University of Bolzano-Bozen, Bolzano, Italy; ^2^Department of Education, University of Bologna, Bologna, Italy; ^3^College of Education, Psychology, and Social Work, Flinders University, Adelaide, SA, Australia; ^4^Department of Psychology, University of Bologna, Bologna, Italy

**Keywords:** cyberbullying perpetration, problematic Internet use, emotional symptoms, family, parental monitoring, adolescence, risk factors, time online

## Abstract

Cyberbullying perpetration (CBP) and problematic Internet use (PIU) are the most studied risky online activities for adolescents in the current generation. However, few studies have investigated the relationship between CBP and PIU. Still lacking is a clear understanding of common or differentiated risk and protective pathways for adolescents interacting in the cyber world. The aim of this study was to understand the role of individual (emotional symptoms) and environmental variables (parental monitoring) underpinning both CBP and PIU, with time spent online as a mediator of these factors. Furthermore, we investigated gender and school level differences in these dynamics. A questionnaire was filled in by 3,602 students from Italian Lower Secondary Schools and Upper Secondary Schools. Structural equation modeling was used to test the effects of emotional symptoms and parental monitoring on CBP and PIU mediated by time spent online, controlling for school level. In addition, the model was implemented for girls and boys, respectively. Negative emotional symptoms and low levels of parental monitoring were risk factors for both CBP and PIU, and their effect was mediated by the time spent online. In addition, parental monitoring highlighted the strongest total effect on both CBP and PIU. Risk and protective pathways were similar in girls and boys across Lower Secondary and Upper Secondary Schools, although there were some slight differences. CBP and PIU are the outcomes of an interplay between risk factors in the individual and environmental systems. The results highlight the need to design interventions to reduce emotional symptoms among adolescents, to support parental monitoring, and to regulate the time spent online by adolescents in order to prevent risky online activities.

## Introduction

Information and communication technologies (ICTs) and social media are changing the way we socially interact, calling for a redefinition and reassessment of social boundaries and the relationships that operate within and around them. The integration of information technology with everyday social life has created a complex phenomenon where social contexts, information channels, and network properties interact. Online and offline contexts represent social worlds placed along a continuum that requires a restructuring and reorganization of relations, nested in a complex system (Wellman, 2004).

The virtual context, in fact, is a crucial scenario to be considered when investigating the dynamics of socialization and communication involved in the construction of views, values, and patterns of behavior that define and influence adolescents’ lifestyles, and, consequentially, their psychological well-being. Beyond the benefits of the Internet and ICT expansion into society, there are many risks that result from their misuse (Livingstone et al., 2011): access to discriminatory and prejudicial content and cyberbullying, pornography, sexting, sextortion, online gambling, and videogame addiction have been reported as emerging and alarming behaviors within the adolescent population (Garaigordobil and Aliri, 2013; Romer and Moreno, 2017; Gainsbury et al., 2018).

Cyberbullying perpetration (CBP) and problematic Internet use (PIU)—the latter defined as an entity of dysfunctional behavioral patterns within the spectrum of impulse control disorders (Kormas et al., 2011; Livingstone and Smith, 2014)—are the most studied risky online activities in the current adolescent population.

To date, the most frequently cited definition of cyberbullying, from Smith et al. (2008, p. 376), is as “an aggressive, intentional act carried out by a group or individual, using electronic forms of contact, repeatedly and over time against a victim who cannot easily defend him- or herself.” This definition was later integrated and revised (Tokunaga, 2010; Slonje et al., 2013), suggesting that the criteria of repetition and power imbalance should be modified. Indeed, a single act can constitute CBP since it may be repeated many times (snowball effect), while the power imbalance in cyberbullying can be described as the presence of different technical abilities with ICTs and anonymity. The prevalence of CBP was highly variable across studies in function of methodological research options (definition of phenomenon, recall periods, age of assessment, country involved, etc.; Brochado et al., 2017; Kowalski et al., 2018). Indeed, as revealed by a recent meta-analysis (Brochado et al., 2017), analyzing the prevalence of cyberperpetration in the last 6 months, the variability across the 21 studies investigated was very high, since the range comprised 1.9 to 79.3%.

Concerning PIU, several studies have outlined the potential addictive properties of the Internet (Griffiths and Parke, 2002), particularly for those adolescents who overly use the Internet, who cannot control their behavior online, and who may develop symptoms of compulsive Internet use (Morahan-Martin and Schumacher, 2000), as well as Internet addiction (Young, 1998) or PIU (Caplan, 2002; Shapira et al., 2003; Gámez-Guadix et al., 2013). Despite a lack of consensus in definition, these symptoms refer to the presence of clinically significant distress or impairment in social, occupational, or other important areas of functioning associated with Internet use (Gámez-Guadix et al., 2013; Bányai et al., 2017), with loss of control over the behavior, conflict (internal and interpersonal), absorption with the Internet, use of the Internet to modify one’s mood, and social withdrawal. Durkee et al. (2012) investigated the prevalence of PIU in 11 countries with a sample of 11,956 adolescents from Austria, Estonia, France, Germany, Hungary, Ireland, Israel, Italy, Romania, Slovenia, and Spain. The highest rate of maladaptive Internet use (18.2%) and pathological Internet use (11.8%) was found in Israel, while the sample’s average was around 7.5%. A meta-analysis by Pontes et al. (2015) reported prevalence rates for PIU between 1 and 18%, with an average prevalence rate of 7.5%.

Given their relevance and growing alarm, literature has underlined the role of individual and contextual risk factors that may be involved in CBP and PIU. Concerning CBP, Cross et al. (2015) pointed out a range of factors at the levels of the individual, family, peers, and the community that may interact with cyberbullying, underlying an ecological framework for understanding this phenomenon. This framework has also been adopted in the systematic review of meta-analyses on protective factors against bullying and cyberbullying by Zych et al. (2019), who reported protective factors against CBP at community, school, family, peer, and individual levels.

At the individual level, research has shown that experiences with cyberbullying as an offender have been associated with significantly lower levels of self-esteem, even while controlling for gender, race, and age (Hinduja and Patchin, 2010; Brighi et al., 2012a; Guarini et al., 2012).

Conversely, Zych et al. (2019) found that a high of level self-esteem, high empathy, and high academic performance were protective factors against CBP. A meta-analysis by Guo (2016) that examined the predictors of CBP at the demographic, individual, and contextual level across 77 studies found that gender was a small to medium predictor of CBP, with higher levels of CBP among males. Age had a relatively small, yet significant, effect size: older students had a significant higher probability of being a cyberbully, but not a cybervictim. This finding was also confirmed by Ybarra (2004) study, since older adolescents were more often cyberbullies than younger ones.

In terms of individual characteristics, the role of internalizing and externalizing problems was analyzed too. The meta-analysis by Guo (2016) showed that internalizing problems had a stronger association with the perpetration of cyberbullying, even if the relationship diminished with the increasing average age of the sample. Strong predictors of CBP included experiencing offline victimization and perpetrating bullying, as well as reporting some externalizing problems. Thus, individuals who were traditional bullies and traditional victims and had been responsible for several delinquent, defiant, aggressive, and rule-breaking acts were more prone to being cyberbullies. Campbell et al. (2013) reported that cyberbullies had more social difficulties and higher scores on stress, depression, and anxiety scales than those students who were not involved in any bullying.

Other authors adopted an ecological approach in investigating PIU risk factors (Anderson et al., 2017; Cacioppo et al., 2019). For what concerns PIU, many studies have reported an association with increased depression and anxiety (Kim et al., 2006; Park et al., 2013; Lee et al., 2014), and personality traits such as impulsivity, hostility, irritability, and lower self-esteem (Cao et al., 2007; Ko et al., 2007, 2015; Yen et al., 2008). These emotional related problems have been reported as being associated with PIU in a recent meta-analysis by Fumero et al. (2018).

The role of the family has been considered in many studies, both as a predictor and as a mediator of adolescents’ use of the Internet. The quality of the affective relationship with parents (Li, 2007) and parental monitoring of young people’s activities online have been found to be inversely associated with CBP (Mesch, 2009; Wade and Beran, 2011; Brighi et al., 2012a; Guarini et al., 2013; Guo, 2016; Melotti et al., 2018; Baldry et al., 2019; Zych et al., 2019). The systematic review of 154 studies by Nocentini et al. (2018) highlighted that parental supervision and monitoring were protective factors for cyberbullying, while the role of overprotective parents was not consistent across studies.

Similarly, recent studies have identified the contribution of attachment style (Cacioppo et al., 2019), family structure, and interactions (Wartberg et al., 2014) in predicting PIU. A recent meta-analysis (Anderson et al., 2017) demonstrated a consistent association between parenting, family-related factors, and levels of Internet use and PIU, particularly in adolescence: good parent–child communication about Internet use was associated with less risk of PIU (van den Eijnden et al., 2010; Yu and Shek, 2013). Paradoxically, parental restriction of online activities (i.e., gaming) did not have a significant impact on PIU levels (Liau et al., 2015). In general, adolescents with closer relationships with their parents showed decreased PIU symptoms over time.

Relevant research has examined possible correlates among risky online activities for adolescents, such as CBP and PIU and the time spent online (Erdur-Baker, 2010; Guo, 2016). The usage frequency of Internet-based communication tools and risky Internet usage were found to be related to both cybervictimization and cyberbullying, even after controlling for the effects of traditional bullying experiences for both male and female students. Adolescents who spend more time on the Internet may expose themselves to a number of potential risks, such as being the target of harassment, invasion of privacy online, identity theft, or sexual exploitation and manipulation (Eksi, 2012; Kırcaburun and Baştug, 2016) and/or may display problematic Internet usage.

Among studies that have described individual and family risk factors as well as the time spent online associated with CBP and PIU, few have investigated the relationship between CBP and PIU (for a meta-analysis, see Kowalski et al., 2014). In particular, CBP has been found to be directly associated with PIU (Keith and Martin, 2005; Eksi, 2012; Casas et al., 2013; Gámez-Guadix et al., 2013; Jung et al., 2014; Nartgün and Cicioğlu, 2015; Kırcaburun and Baştug, 2016). This relationship was found not only in cross-sectional studies but also in longitudinal studies where cyberbullying victimization predicted PIU 6 months later (Gámez-Guadix et al., 2013). At the same time, PIU was a significant predictor of CBP, with the amount of time people spent on the Internet often being linked with cyberbullying behavior (Kırcaburun and Baştug, 2016; Kırcaburun et al., 2018).

However, to our best knowledge, none of them adopted this ecological approach in investigating both CBP and PIU at the same time as outcomes; moreover, as emphasized by Zych et al. (2019), most of the studies in the field have not differentiated between risk factors and different types of protective factors against cyberbullying. Indeed, according to Zych et al. (2019), a factor that can be protective can, at the same time, be a risk factor. Therefore, a deeper knowledge of protective factors against CBP and PIU should be gained.

In accordance with the ecological framework, our study sought to investigate two main levels of the ecological system such as individual factors (emotional symptoms) and environmental factors (parental monitoring) on PIU and CBP, taking into account time spent online as mediator. The amount of time spent online, in fact, appears to be related to both parental monitoring behaviors (Khurana et al., 2015) and emotional symptoms (Cao et al., 2011), and was found to be one of the most important factor associated to PIU and CBP (Erdur-Baker, 2010; Guo, 2016). In our model, controlling for school level, emotional symptoms and parental monitoring were considered potential risk and protective factors for CBP and PIU, with time spent online partially mediating their effects on CBP and PIU. In particular, we hypothesized that emotional symptoms and parental monitoring would have both a direct effect on CBP and PIU and an indirect effect mediated by the influence of time that young people spent online. In addition, since literature has outlined that risk and protective factors can be differently modulated across genders (Guo, 2016), the proposed model was tested among male and female groups. Our study accounted for both risk and protective factors at individual and family levels, by exploring both their direct relation and their mediated relation with CBP and PIU, thus contributing to advance the knowledge regarding the way young people can be protected against these phenomena.

## Materials and Methods

### Participants

A two-stage, non-probabilistic sampling method was applied in order to approximate a representative sample of the students in the Emilia Romagna region of Italy (for more information, see Guarini et al., 2013). Following the sampling procedure, 61 Secondary Schools were enrolled, comprising 16 Lower Secondary Schools and 45 Upper Secondary Schools, including Lyceum, technical institutes, and vocational institutes.

The survey was completed in 2014–2015 by 3602 students (56% were males, *n* = 2010), including Lower Secondary School students (*n* = 934, 26%) and Upper Secondary School students (*n* = 2668, 74%). Students’ ages ranged from 11 to 20 years (*M* = 14.64, *SD* = 1.70). Students with non-Italian citizenship represented 17.1% of the sample (21% in Lower Secondary Schools and 15.4% in Upper Secondary Schools).

A combined analysis of the level of education of both parents showed that 23.5% of students had one parent who completed Primary or Lower Secondary School, 12.2% had both parents who completed Lower Secondary School, 50.3% had at least one parent with Upper High School or University degree, while 14.5% had both parents with this educational level. Most participants (79.7%; *n* = 2872) reported living with both parents, while 17.8% of students were from single-parent households.

### Measures

Participants completed an anonymous, self-report questionnaire based on the European Cyberbullying Intervention Project ECIP questionnaire (ECIPQ, Brighi et al., 2012b; Del Rey et al., 2015). The questionnaire was translated and validated into five different languages (Schultze-Krumbholz et al., 2015), and for this reason, it was chosen among validated tools for the Italian population. It included the following sections.

#### Participant Information

Sociodemographic information, such as gender, age, and parents’ education, was collected in this section.

#### Cyberbullying Perpetration

Cyberbullying was assessed using the Italian version of the cyberbullying scale from the European Cyberbullying Intervention Project Questionnaire (ECIPQ, Brighi et al., 2012b; Del Rey et al., 2015; Schultze-Krumbholz et al., 2015).

The CBP scale consists of seven items (e.g., “I threatened someone with messages on the Internet” and “I threatened someone by using SMS”). Participants were asked to evaluate their experiences of CBP in the last 6 months, using a five-point scale (never, once or two times, two or three times per month, once a week, more times a week). The CBP scale displayed a good reliability, as coefficient *H* (Hancock and Mueller, 2001) was 0.792.

#### Problematic Internet Use

An adapted and reduced version of the subscale “NCT Engagement” was included in the Lodz Electronic Aggression Prevalence Questionnaire (Pyżalski, 2009) and was used in order to measure PIU. The Italian version of the scale was validated for the Italian population in the ECIPQ (Brighi et al., 2012b; Guarini et al., 2013). The five items were included, as indicators of Internet use, which could be considered problematic (“I get bored if I cannot connect to internet,” “On days when I’m free, I spend all my time at the computer,” “Better that no one knows what I do on the computer,” “Often I don’t sleep during the night because I’m using the computer,” “I feel better in virtual world than in the real world”). Participants responded to these questions as true (1) or false (0). A PIU score was computed, using the sum of the five items (ranging from 0 to 5). The factor was found to be quite reliable (coefficient *H* = 0.797).

For descriptive purposes, PIU scores were used to divide participants into four groups: “Not Evident” (with a score of zero), “Low Level” (with a score of one), “Medium Level” (with a score of two), and “High Level” (with a score of three or more).

#### Online Time

Online time was assessed using the Italian version of a three-item scale from the ECIPQ (Brighi et al., 2012b; Guarini et al., 2013). The three items were, respectively, “How long do you use internet in a normal working day?,” “How long do you play videogames in a normal working day?,” and “How long do you use technological tools in a normal working day?” Participants responded to the questions choosing the time that was more indicative of their use of Internet, from “less than 20 min a day” to “more than 5 h a day.” The reliability of the online time scale displayed a coefficient *H* of 0.663.

#### Emotional Problems

For this study, the Emotional Symptoms Subscale of the SDQ (Goodman, 2001) was adopted, since the Italian validated version of the scale was available (Di Riso et al., 2010). The five items were, respectively, “I get a lot of headaches, stomach-aches, or sickness,” “I worry a lot; I am often unhappy, depressed or tearful,” “I am nervous in new situations,” “I easily lose confidence,” and “I have many fears, I am easily scared.”

Each item was scored on a three-point scale with 0 = “not true,” 1 = “somewhat true,” and 2 = “certainly true.” The Subscale score was computed by summing the scores on the five items (range = 0 to 10). Coefficient *H* for the scale was 0.714, indicating an acceptable reliability. For descriptive purposes, we applied the categorization by Goodman (1997) for the Emotional Symptoms subscale of the SDQ scale, summing the numeric scores (0–2) of the five items and coding the scores as normal (<4), borderline (=4), and abnormal (>4).

#### Parental Monitoring

A reduced and adapted version of the Parental Monitoring of Internet activities scale, validated in the ECIP questionnaire (Brighi et al., 2012b) and originally developed by Law et al. (2010), was used to assess parental monitoring. This included five items about the relationships with parents concerning Internet use (e.g., “Do your parents give you a time limit that you can spend on Internet?,” “Do your parents really know what you do on Internet and which sites you visit?”). Students responded on a Likert scale ranging from 0 (never) to 5 (almost ever). The items covered not only the dimension of parental control (i.e., setting rules and time limits for Internet use, knowing what their child is doing online, soliciting information from their children) but also the child’s will to disclose to parents his/her experiences online (Stattin and Kerr, 2000). The internal reliability of the Parental Monitoring scale was good, with coefficient *H* being 0.821.

### Procedure

The online anonymous self-report questionnaire was administered in ICT classes. A trained researcher was present during the administration, in order to respond to possible questions. Students who were not allowed to take part in the study were involved in other activities carried on by class teachers. The questionnaire took about 30 min to complete. A researcher was available to provide explanations for students who may have had linguistic problems.

### Ethics Statement

The study protocol met the ethical guidelines for the protection of human participants, including adherence to the legal requirements of Italy, and received a formal approval from the Bioethics Committee, University of Bologna. School directors and teachers were informed about the project. Parents provided their informed written consent for allowing the participation of their son/daughter in the study. Students were also informed about the survey’s procedure and aims and were given the opportunity to refrain from participation with no negative consequences.

### Data Analysis

We used confirmatory factor analysis (CFA) to test the measurement model and structural equation modeling (SEM) to investigate the potential mediation of time spent online in relation to emotional symptoms and parental monitoring on the one hand, and PIU and cyberbullying (CBP) on the other.

Goodness of fit was assessed using various fit indexes, namely, the comparative fit index (CFI), the Tucker–Lewis index (TLI), the root mean square error of approximation (RMSEA) and corresponding 90% confidence interval, and the standardized root mean square residual (SRMR). CFI and TLI values above 0.90 and RMSEA and SRMR values lower than 0.06 and 0.08, respectively, were considered indicative of an acceptable fit (Hu and Bentler, 1999; Kenny, 2015).

The weighted least squares with means and variance adjusted (WLSMV), a robust version of the diagonally weighted least squares (DWLS) method, was adopted for parameter estimation, in order to accommodate for the ordinal nature of our data (Beauducel and Herzberg, 2006; Rhemtulla et al., 2012; Li, 2016), and standardized coefficients were used. Analyses were carried out using Lavaan version 0.5-23.1097 in R version 3.4.3.

## Results

### Descriptive Statistics

Among cyberbullying behaviors, “I have told others some unpleasant things about someone else online” was the most commonly reported behavior (see Table 1), having been displayed once or twice over the last 6 months by 21.6% of respondents and at least once a month by 4.5% of respondents. Other forms of cyberbullying were less frequent even if serious in terms of their consequences on victims. For example, 9.3% of students (*n* = 331) admitted to having violated someone else’s account at least once while 8.5% (*n* = 303) had created a fake account pretending to be someone else in the past 6 months.

**TABLE 1:** Participants’ cyberbullying perpetration.

**Item**	**Never**		**Once or twice**		**At least once a month**	
	**Count**	**%**	**Count**	**%**	**Count**	**%**
(1) I said unpleasant things or I offended someone online	2,791	78.31	626	17.56%	147	4.12
(2) I have told others some unpleasant things about someone else online	2,613	73.90	764	21.61%	159	4.50
(3) I have violated someone else’s account	3,226	90.69	255	7.17%	76	2.14
(4) I created a fake account pretending to be another person	3,254	91.48	250	7.03%	53	1.49
(5) I posted embarrassing pictures or videos online	3,352	94.18	149	4.19%	58	1.63
(6) I have excluded or ignored someone on social networks	2,877	80.93	503	14.15%	175	4.92
(7) I attacked or insulted someone in a game	2,971	83.62	270	7.60%	312	8.78

**N* = 3,602.*

At least one type of PIU behavior was reported during the past 6 months by more than half of the sample (*n* = 2,024, 57.6%). As displayed in Table 2, among the different behaviors, “I get bored if I cannot connect to the internet” was the most common response (*n* = 1370, 39.1%), followed by “It’s better that no one knows what I do on the computer” (*n* = 843, 24.1%).

**TABLE 2:** Descriptive (count and percentages) of PIU.

**Index**	**Total sample**		**Low PIU**		**Medium PIU**			**High PIU**	
	**Count**	**%**	**Count**	**%**	**Count**	**%**	**Count**		**%**
(1) I get bored if I cannot connect to the Internet	1,370	39.09	470	48.91	405	78.95	469		91.78
(2) In days when I’m free, I spend all my time on the computer	601	17.17	73	7.60	158	30.80	349		68.30
(3) It’s better that no one knows what I do on the computer	843	24.13	261	27.16	216	42.11	356		69.67
(4) I often don’t sleep during the night because I’m on the computer	430	12.31	52	5.41	101	19.69	271		53.03
(5) I feel better in a virtual world than in the real world	596	17.11	105	10.93	146	28.46	334		65.36

**N* = 3,602.*

The total PIU scores were used to classify participants into four groups: no signs of PIU (score = 0; *n* = 1469) appeared for 42.5% of respondents, low PIU level (score = 1; *n* = 961) was observed in 27.8%, medium PIU level (score = 2, *n* = 513) was observed in 14.9% of the participants, and high PIU level (score of 3 or more, *n* = 511) was found for 14.8%.

Participants with a low PIU level were most likely to affirm that they were bored if they could not connect to the Internet (*n* = 470, 48.9%), while just over one-quarter (*n* = 261; 27.2%) thought that it was “Better that no one knows” what they did on the computer. Only a small proportion (*n* = 105, 10.9%) of participants in this group reported that they felt better in the virtual rather than the real world.

Participants with medium PIU level reported feeling bored without the Internet (*n* = 405, 78.9%) and that it would be better that no one knows what they did on the Internet (*n* = 216, 42.1%). However, less than one-third (*n* = 158, 30.8%) reported that they spent most of their free time on the Internet, while just over one-quarter (*n* = 146, 28.5%) reported feeling better in the virtual rather than the real world and one-fifth (*n* = 101, 19.7%) indicated that they did not sleep because they were using the computer.

Nearly all participants with high PIU level reported feeling bored without the Internet (*n* = 469, 91.8%). In addition, approximately two-thirds indicated that they spent most of their free time on the Internet (*n* = 349, 68.3%), felt better in the virtual rather than the real world (*n* = 334, 65.4%), and that it would be better that no one knows what they did on the Internet (*n* = 356, 69.7%). About half (*n* = 271, 53.0%) of the participants reported that they did not sleep because they were using the computer.

Concerning online time, results highlighted that exposure to Internet varied, as 30.6% of respondents (*n* = 1,054) reported spending less than 1 h in a normal working day, 45.5% (*n* = 1,564) spent from 1 to 3 h a day on Internet activities, while 23.9% (*n* = 822) browsed the Internet from at least 3 h a day to more than 5 h (see Table 3 for detailed incidences).

**TABLE 3:** Participants’ report of parental monitoring, emotional symptoms, and online time.

**Parental monitoring**						
**Item**	**Never/rarely**		**Sometimes**		**Often/always**	
	**Count**	**%**	**Count**	**%**	**Count**	**%**
(1) Do your parents really know what you do when you surf on the Internet and what sites you visit?	1,063	29.86	848	23.82	1,649	46.32
(2) How often do you tell your parents what you and your friends do when you’re on the Internet?	2,014	56.51	835	23.43	715	20.06
(3) Do you have to tell your parents what you’re doing on the Internet?	2,387	67.58	591	16.73	554	15.69
(4) How often do your parents talk to you about what you’re doing on the Internet?	2,324	65.99	718	20.39	480	13.63
(5) Do your parents give you a limit on the time that you spend on the internet and sites that you can visit?	2,070	58.41	664	18.74	810	22.86
**Emotional symptoms**						
**Item**	**Not true**		**Partially true**		**Totally true**	
	**Count**	**%**	**Count**	**%**	**Count**	**%**

(1) I get a lot of headaches, stomach aches, or sickness	2,185	61.78	953	26.94	399	11.28
(2) I worry a lot	814	23.20	1690	48.16	1,005	28.64
(3) I am often unhappy, down-hearted, or tearful	2,140	61.71	959	27.65	369	10.64
(4) I am nervous in new situations. I easily lose confidence	1,414	40.52	1546	44.30	530	15.19
(5) I have many fears, I am easily scared	2,160	61.66	1021	29.15	322	9.19
**Online time**						
**Item**	**Less than 1 h**		**1–3 h**		**More than 3 h**	
	**Count**	**%**	**Count**	**%**	**Count**	**%**

(1) How long do you use Internet in a normal working day?	1,054	30.64	1564	45.47	822	23.90
(2) How long do you play video games in a normal working day?	1,918	61.63	944	30.33	250	8.03
(3) How long do you use technological tools in a normal working day?	780	23.53	1546	46.64	989	29.83

**N* = 3,602.*

In terms of Emotional Symptoms, 55.5% of the sample (*n* = 1,998) was coded as normal (score < 4), 11.9% (*n* = 430) as borderline (score = 4), and 26% (*n* = 947) as abnormal (score > 4), while 6.3% (*n* = 227) was not categorized due to missing values.

Regarding parental monitoring, the survey highlighted a rather diversified situation. In particular, 46.3% of respondents (*n* = 1649) reported their parents were often or always aware of their online activities while 35.7% (*n* = 1256) were never talked to by their parents in relation to online behavior. Moreover, 58.4% of respondents reported that their parents never or rarely gave them a time limit for surfing the Internet.

### CFA and SEM

The five-factor CFA model, including emotional symptoms, parental monitoring, online time, CBP, and PIU, showed a good fit with the data: CFI = 0.919, TLI = 0.909, RMSEA = 0.048 (90% CI 0.046; 0.050), SRMR = 0.068. Factor loadings were significantly (*p* < 0.001) different from zero for each measured variable, confirming the goodness of the measurement model and its factorial structure.

As shown in Table 4, the CFA highlighted significant covariance (with *p* < 0.001) between all the study variables, in the expected directions. Intraclass correlation (ICC) coefficients were checked to determine whether multilevel modeling was needed. Since all ICC coefficients were very low (<0.06), we concluded that single-level analyses were appropriate (Byrne, 2006; Gámez-Guadix et al., 2013).

**TABLE 4:** CFA covariance matrix.

	**PIU**	**CBP**	**PM**	**OT**
**Problematic Internet use (PIU)**				
Cyberbullying (CBP)	0.422^∗∗∗^			
Parental monitoring (PM)	–0.416^∗∗∗^	–0.286^∗∗∗^		
Online time (OT)	0.589^∗∗∗^	0.398^∗∗∗^	–0.264^∗∗∗^	
Emotional symptoms (ES)	0.262^∗∗∗^	0.131^∗∗∗^	0.145^∗∗∗^	0.101^∗∗∗^

*^∗∗∗^*p* < 0.001.*

In addition, in order to exclude multicollinearity between online time and PIU, as their covariance was relatively high (β = 0.589, *p* < 0.001), a four-factor CFA model was also fitted, with the items from these two constructs being loaded onto the same latent variable. This reduced model yielded consistently worse fit indexes than the full model: CFI = 0.904, TLI = 0.893, RMSEA = 0.052 (90% CI 0.050; 0.054), SRMR = 0.073. The full five-factor model was therefore retained for SEM analysis.

The hypothesized model was then fitted to the whole dataset, with emotional symptoms and parental monitoring as exogenous variables, online time as mediator and PIU and CBP as outcomes, while the effect of school level (Lower vs. Upper Secondary School) was controlled on all study variables.

**FIGURE 1:**
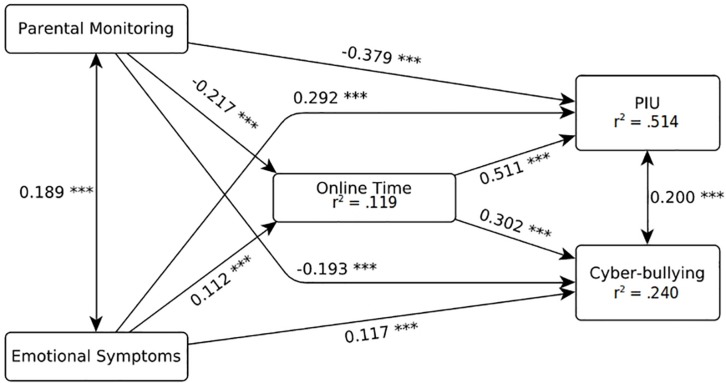
SEM model fitted on the whole dataset. ^∗∗∗^*p* < 0.001.

Model fit indexes indicated that the model fit well with the data, CFI = 0.939, TLI = 0.946, RMSEA = 0.035 (90% CI 0.033; 0.037), SRMR = 0.068. As shown in Figure 1, all effects were significant (with *p* < 0.001). In particular, online time was negatively predicted by parental monitoring (β = −0.217, *p* < 0.001) and positively—although more weakly—by emotional symptoms (β = 0.112, *p* < 0.001). CBP was negatively predicted by parental monitoring (β = −0.193, *p* < 0.001) and positively predicted by emotional symptoms (β = 0.117, *p* < 0.001) and online time (β = 0.302, *p* < 0.001). PIU highlighted a similar pattern, being positively predicted by online time (β = 0.511, *p* < 0.001) and—more weakly—by emotional symptoms (β = 0.292, *p* < 0.001) and negatively predicted by parental monitoring (β = −0.379, *p* < 0.001).

In general, the effects of both emotional symptoms and parental monitoring on CBP and PIU were partially mediated by online time. Parental monitoring, in particular, highlighted the strongest total effect on both CBP (β = −0.258, *p* < 0.001) and PIU (β = −0.490, *p* < 0.001), with the mediation of online time accounting for 26% (β = −0.066, *p* < 0.001) and 23% (β = −0.111, *p* < 0.001), respectively, of total effects (see Table 5). Emotional symptoms showed weaker total effects on CBP (β = 0.151, *p* < 0.001) and PIU (β = 0.349, *p* < 0.001), with mediation of online time accounting for 23% (β = 0.034, *p* < 0.001) and 16% (β = 0.057, *p* < 0.001) of total effects, respectively. Residual covariance was significant both between CBP and PIU (β = 0.200, *p* < 0.001) and between parental monitoring and emotional symptoms (β = 0.189, *p* < 0.001). In addition, all of the study variables were affected by school level, with Upper Secondary students being more at risk for cyberbullying (β = 0.149, *p* < 0.001) and emotional symptoms (β = 0.107, *p* < 0.001), less at risk for PIU (β = −0.201, *p* < 0.001) and reporting more time online (β = 0.191, *p* < 0.001), as well as less parental control (β = −0.304, *p* < 0.001).

**TABLE 5:** Direct, indirect, and total effects of parental monitoring and emotional symptoms on CB and PIU.

**Outcome**	**Predictor**	**Direct**	**Indirect**	**Total**
		**effect**	**effect**	**effect**
CBP	Parental monitoring	–0.193	–0.066	–0.258
	Emotional symptoms	0.117	0.034	0.151
PIU	Parental monitoring	–0.379	–0.111	–0.49
	Emotional symptoms	0.292	0.057	0.349

*The table reports fully standardized coefficients; CBP, cyberbullying; PIU, problematic Internet use.*

**FIGURE 2:**
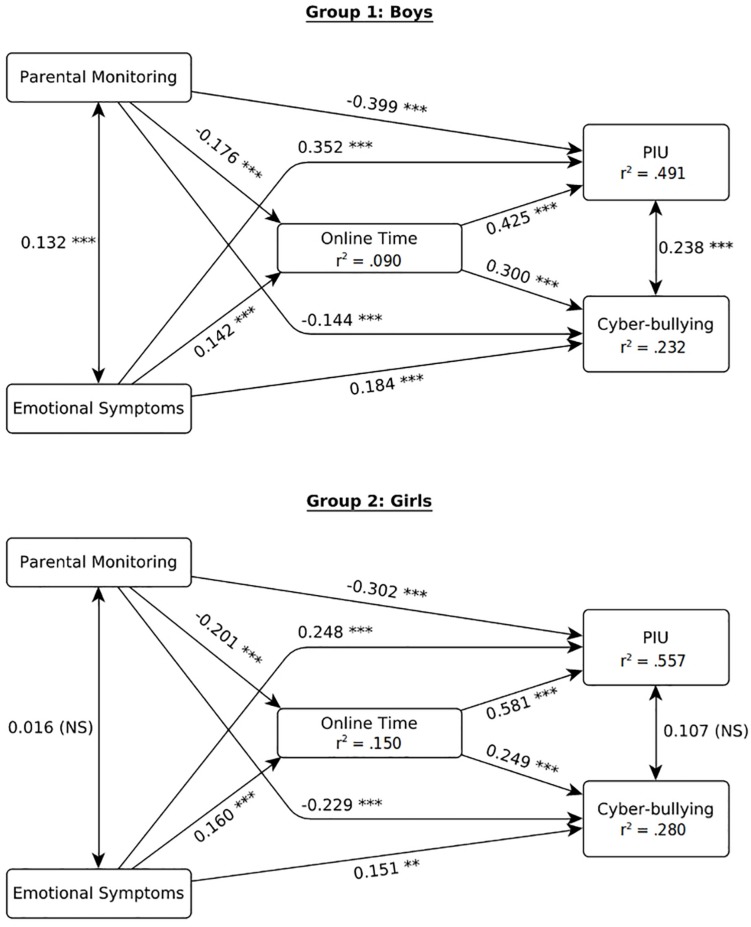
SEM model fitted with gender as grouping variable. ^∗∗∗^*p* < 0.001; ^∗∗^*p* < 0.01; ^*^p < 0.05; NS *p* ≤ 0.05.

In order to investigate gender differences, a second model was fitted, using the same formula, accounting for school level and adopting gender as grouping variable. This model highlighted a consistently better fit, CFI = 0.953, TLI = 0.958, RMSEA = 0.029 (90% CI 0.028; 0.031), SRMR = 0.064. As shown in Figure 2, results highlighted overall similar effects across gender (with *p* < 0.001 for all regressions). However, among males, there was a stronger direct effect of parental monitoring (males: β = −0.399, *SE* = 0.041; females: β = −0.302, *SE* = 0.040) and emotional symptoms (males: β = 0.352, *SE* = 0.052; females: β = 0.248, *SE* = 0.044) and a weaker effect of online time (males: β = 0.425, *SE* = 0.044; females: β = 0.581, *SE* = 0.057) on PIU compared to females. As a consequence, the mediation of online time accounted for a bigger portion of the effects of both parental monitoring and emotional symptoms on PIU in females (27.9% for parental monitoring and 27.3% for emotional symptoms) compared to males (15.8 and 14.6%, respectively).

School level held similar effects for males and females, with the exception of emotional symptoms, which were more severe in Upper Secondary School compared to Lower Secondary School for girls (β = 0.267, *p* < 0.001) while not being affected by school level for boys (β = −0.018, *p* = 0.567).

Moreover, males highlighted significant residual covariances both between CBP and PIU (β = 0.238, *p* = 0.001) and between parental monitoring and emotional symptoms (β = 0.132, *p* < 0.001), while no residual covariance was significant for females. Consistently, the model accounted for slightly more variance in females, resulting in higher *R*^2^ values for online time (*R*^2^ = 0.150), cyberbullying (*R*^2^ = 0.280), and PIU (*R*^2^ = 0.557) compared with males (*R*^2^ = 0.090, *R*^2^ = 0.232, and *R*^2^ = 0.491, respectively).

## Discussion

Our study sought to investigate the role of emotional symptoms and parental monitoring of online activities on CBP and PIU, taking into account the time spent online as a mediator. This study presents an important element of innovation, since it considers both CBP and PIU as outcomes of common risk pathways, within an ecological framework by exploring the contribution of individual and contextual factors.

According to our results, both CBP and PIU are behaviors with a worrisome diffusion among Italian adolescents. Concerning CBP, about one-quarter of the adolescents admitted some forms of CBP. Our data confirmed high involvement in cyberbullying among Italian students, as already described in previous studies in Europe (Genta et al., 2012; Del Rey et al., 2015). Concerning PIU, indexes of serious PIU were displayed by about 30% of the adolescents, with some signs of an addictive relationship with communication technologies. These results are alarming, since the result from a meta-analysis reported prevalence rates of PIU from 1 to 18%, with an average rate around 7.5% (Pontes et al., 2015). In our sample, high exposure to the Internet was observed, with almost a fourth of the sample spending more than 3 h online per working day. This result is consistent with a study carried out in 2017 involving preadolescents and adolescents in Italy (IPSOS, 2017) that showed that about one-third of teenagers are connected for more than 5 h a day.

Concerning individual and contextual factors, more than half of respondents did not report any kind of parental monitoring over their online activity, depicting an image of distance between parents and children in reference to what happens on the Internet. Almost one-third of adolescents reported negative emotional symptoms. This result is in line with HBSC and PISA national Italian surveys (Cavallo et al., 2015; OECD, 2017), where almost one-third of students were found to have two or more psychosomatic symptoms (Cavallo et al., 2015). Higher scores for stress, anxiety, and psychosomatic symptoms were reported in the Italian sample, compared to students from other European Countries (OECD, 2017).

Analyzing how emotional symptoms and parental monitoring of online activities could be connected to CBP and PIU, and as problematic outcomes of the interplay between individual and contextual factors, our results highlighted that negative emotional symptoms and a lack of parental monitoring both had a direct effect and an indirect effect, mediated by time spent online, on CBP and PIU, increasing the risk for both of them. However, it is worth noting that time online alone was not a sufficient risk factor for CBP and PIU as its mediation explained only about one-fourth of the effects. It increased, instead, the risk, starting from a situation of vulnerability. Time online, in fact, seemed to add further risk, in the framework of a general underlying risky configuration, where high emotional symptoms and lack of parental monitoring depicted a scenario of potential vulnerability to CBP and PIU.

The direct and mediated effects of negative emotional symptoms on CBP and PIU can be explained by the assumptions made by De Leo and Wulfert (2013) who suggested that PIU appeared to be related to internalizing behavioral problems, such as depression and social interaction anxiety. In our results, this seems true also for CBP that shares with PIU the same risk pathways, confirming findings reported by Guo (2016). This assumption was confirmed by the longitudinal study by Gámez-Guadix et al. (2013) who demonstrated that adolescents who are bully/victims of cyberbullying were more likely to develop depressive symptoms (Orth et al., 2008; Kırcaburun and Baştug, 2016) and PIU.

The association between emotional symptoms and online risky behaviors can be explained by the Social Compensation Theory (Valkenburg and Peter, 2009). Internet may be used to reduce anxiety, feelings of isolation, or negative emotions (Caplan, 2002; Gámez-Guadix et al., 2012) and can provide an easy access to dealing with suppressed anger, aggression, and hostility (Gackenbach, 2011; Fumero et al., 2018). In addition, the Internet may represent a way of coping with life difficulties, taking the form of problem solving through avoidance. The anonymous environment of the cyber world may lead to the psychological effect termed as “online disinhibition effect” (Yen et al., 2008; Ko et al., 2014) as a predisposing factor of abusive Internet use and for CBP. Thus, PIU and CBP can be conceptualized as a form of maladaptive self-regulatory strategy (Spada et al., 2008; Spada, 2014). This was supported by the mediation exerted by time spent online.

Concerning parental monitoring, our study confirms that this may play an important role in CBP and PIU, partly through a control of the amount of time that adolescents spend online and also by parents soliciting information from their children about their activities online. The construct of parental monitoring adopted in our study included not only the role of control but also items pertaining more to the quality of the dialogue with parents around Internet issues. Parental monitoring, in fact, has been conceptualized as a multidimensional construct, not limited to the dimension of control, but also including adolescents’ disclosure and parental trust, which is embedded and develops in a two-way relational process between parents and children (Stattin and Kerr, 2000). Indeed, parental bonding with children inhibits problematic behavior and serves as a protective factor for adolescent problematic behaviors (Liu et al., 2013), both offline and online (Brighi et al., 2012a). The relationship among parental monitoring and CBP has been consistently confirmed in literature, although the specific dimensions of parental monitoring could exert different influences on children’s behavior, as highlighted by research on adolescent’s deviant behaviors. Melotti et al. (2018) showed that adolescents reporting low control, low trust, and low disclosure were involved more often in physical, psychological, and CBP compared to groups not at risk for these behaviors. This result has been confirmed for CBP also by Law et al. (2010) and Brighi et al. (2012a) who demonstrated that low trust and low child disclosure were more predictive of CBP than high control itself. Thus, given the strict relation between CBP and PIU with parental monitoring reported in our study, it could be relevant for future research to investigate the role of different dimensions of parental monitoring that may differently be related to risky behaviors online.

It may also be relevant to consider how flexible parental monitoring should be across development, according to the developmental needs of preadolescents and adolescents, because a dimension such as control could be a protective factor at a young age, while it may play a negative role when the requests for autonomy increase during later adolescence. In this regard, the suggestion made by Zych et al. (2019), to also consider as protective factors those variables that can be protective and risky at the same time, depending on their intensity and timing, seems particularly relevant. Therefore, parental monitoring can be a protective factor against CBP and PIU when it is balanced between control and openness, and is adequate for the child’s self-regulation competencies, while it may act as a risk factor when it is low or over-controlling.

Concerning possible differences between males and females in the effect of emotional symptoms and parental CBP and PIU, our study suggested similar patterns in functioning of gender, even if two slight differences were found. First, the model explained a higher portion of the variability both for CBP and PIU among females, highlighting for boys a significant residual covariance between CBP and PIU. This result supports the claim that the proposed model accounts for most of the relationship between CBP and PIU, at least for female adolescents. On the other hand, additional shared risk pathways, such as externalizing symptoms, could contribute in a relevant way to the manifestation of CBP and PIU in male adolescents. Second, the portion of un-mediated effects of emotional symptoms and parental monitoring on PIU was higher in the male group compared to the female sample.

Our evidence suggests that most of the impact of both emotional symptoms and parental monitoring on the risks of becoming a cyberbully or developing PIU is not due to the associated increase or reduction of online time. Adolescents (boys in particular) seem to benefit more from parental monitoring in terms of a reduction in risk than indicated by the decrease in the time they spend online, confirming the importance of establishing a dialogue between parents and adolescents on the topics of online environments and behaviors.

A specular situation can be described for emotional symptoms, whose disruptive effects in terms of risky online behavior cannot be counterbalanced by simply reducing the time adolescents spend online. These results are consistent with the hypothesis that CBP and PIU may share complex multi-level risk and protective pathways, both at individual and contextual levels. Moreover, the differentiation of risk pathways between males and females suggests that different populations might be variously sensitive to different risk factors and mediators.

Although the present study suggests that individuals experiencing mood disruptions or family lack of control and of dialogue may be at greater risk of developing PIU and CBP, the link between PIU and CBP still lacks a conceptual framework that could explain it. In addition, the residual covariance of the proposed model remains high (at least for boys), so it would be important to investigate further underlying factors—both at individual and at contextual levels—that may help to disentangle the relationship between the two risky online behaviors. Therefore, it is important to further develop an understanding of other relevant factors that are associated with PIU and CBP, which might act to influence the relative salience of Internet use as a reinforcing agent in the environment. Future research, for example, might identify those cognitive distortions about the self that accompany pathological Internet behavior and those linked with motivational states that provoke CBP.

Cognitions about the self may include such thoughts as “I am only good on the Internet,” “I am worthless offline, but online I am someone,” and “I am a failure when I am offline” (Davis, 2001). Motivational states could be described as “People treat me badly offline.” These kinds of thoughts have been considered to be maladaptive cognitive distortions that exacerbate the individual’s Internet dependence. These distortions of thought are automatically enacted whenever a stimulus associated with the Internet is available, fueling through gratification an increase of negative behaviors online (Davis, 2001). Thus, similarly to PIU, CBP may also be the result of cognitive distortions and as such could benefit from cognitive behavioral intervention, tackling both representations about the self and motivations for PIU and CBP.

The findings should be considered in light of several limitations. First, we used only self-report questionnaires; thus, it is possible that some participants misunderstood questions or underreported socially undesirable behaviors. Although we adopted all the possible precautions in order to ensure confidentiality and anonymity of the respondents, it is possible that their responses were influenced by social desirability. Second, our study adopted a transversal design, allowing us to depict a picture of the relationship among the variables considered significant in previous studies. However, in order to make appropriate assumptions about cause–effect relationships among variables, a longitudinal investigation would have been more appropriate and fruitful.

While it is clear that problem behaviors typically emerge from an interplay of individual and environmental contexts, more research is needed to identify what factors in the offline context may play a role in the online experience. Indeed, as revealed by several studies, the strongest risk factor for CBP is school bullying (Baldry et al., 2016). In line with the suggestions of De Leo and Wulfert (2013), we support the idea of extending the definitions of “problematic behaviors” to both online and offline individual factors (e.g., motivational) and contextual considerations. Further research should continue to focus on the intersection between how individuals effectively regulate and manage both their online and offline experiences. Finally, assuming an ecological framework (Baldry et al., 2016), in the present study, only emotional symptoms, parental monitoring, and time spent online were taken into account, while other factors and mediators, at individual (self-esteem, moral disengagement), interpersonal (peer support), and community levels (school policy), could explain CPB and PIU phenomena. In addition, Internet-specific approaches (Davis, 2001) aimed at exploring cognitive and motivational distortions (e.g., “I am worthless offline, but online I am someone”; Davis, 2001), potentially associated with pathological Internet behavior and to its problematic uses, could be helpful in tackling self-reinforcing schemas that fuel both aggressive behaviors online and an addictive–compensative use of Internet.

## Conclusion

Our study confirmed negative emotional symptoms and low parental monitoring as risk factors for CBP and PIU, with a mediation role of the time spent online, suggesting several implications for educational interventions aimed at preventing and contrasting PIU and CBP. In particular, our results suggest the need to promote prevention programs for all parents, in order to foster a sensitive but coherent parental monitoring of adolescents’ activities online, wherein the control of adolescents’ activities is accompanied by communication about their experiences online. Improving the quality of dialogue among parents and children would be a means to strengthen a crucial protective factor also for emotional symptoms. At the same time, the intervention for adolescents should focus on developing a responsible and self-regulated use of the Internet, a sort of “internalization of parental monitoring,” helping students to learn how to monitor themselves, while offering additional attention to potential emotional symptoms.

Moreover, the link between emotional maladjustive functioning and PIU/CBP points out to the necessity to tackle some specific types of emotion regulation deficiencies, i.e., awareness, management, and coping, that may underlie internalizing and externalizing symptoms. Deficits in these areas, in fact, have been hypothesized to underlie adolescents’ risk for both internalizing (e.g., anxiety and depression) and externalizing (e.g., oppositional defiance and aggression) problems (Cole et al., 1994), with specific emotional regulation strategies interacting in predicting emotional outcomes. Indeed, literature has shown that adaptive strategies such as reappraisal and acceptance of emotion-eliciting situations were associated with reduced psychopathology symptoms among those who used maladaptive strategies such as rumination, suppression, and avoidance (Aldao and Nolen-Hoeksema, 2012; Webb et al., 2012). This indication suggests that treatments focusing on developing effective and adaptive emotional regulation strategies may also be effective in reducing maladjusted compensatory uses of the Internet among adolescents.

## Data Availability

The datasets generated for this study are available on request to the corresponding author.

## Ethics Statement

This study was carried out in accordance with the recommendations of the Ethical Committee of the University of Bologna with written informed consent from all subjects. All subjects gave written informed consent in accordance with the Declaration of Helsinki. The protocol was approved by the Ethical committee of the University of Bologna on November, 26th, 2012.

## Author Contributions

AB and AG contributed to the conception and design of the study. AG managed the coordination of the research activity and the acquisition of the financial support for the project and assisted in the interpretation of the results. GS performed the statistical analysis and assisted in the interpretation of the results. AB and DM wrote the first draft of the manuscript. DM organized the database and performed the statistical analysis. All authors wrote sections of the manuscript, contributed to its revision, and read and approved the submitted version.

## Conflict of Interest Statement

The authors declare that the research was conducted in the absence of any commercial or financial relationships that could be construed as a potential conflict of interest.
